# Cellulase Promotes Mycobacterial Biofilm Dispersal in Response to a Decrease in the Bacterial Metabolite Gamma-Aminobutyric Acid

**DOI:** 10.3390/ijms25021051

**Published:** 2024-01-15

**Authors:** Jiaqi Zhang, Yingying Liu, Junxing Hu, Guangxian Leng, Xining Liu, Zailin Cui, Wenzhen Wang, Yufang Ma, Shanshan Sha

**Affiliations:** 1 Department of Biochemistry and Molecular Biology, Dalian Medical University, Dalian 116044, China; zjq2901zjq@163.com (J.Z.); 15247578016@163.com (Y.L.); junxinghu2022@163.com (J.H.); 15114101682@163.com (G.L.); xining_liu2002@163.com (X.L.); kimuqi@outlook.com (Z.C.); 17889736446@163.com (W.W.); 2 Department of Microbiology, Dalian Medical University, Dalian 116044, China

**Keywords:** biofilm, biofilm dispersal, *Mycobacterium tuberculosis*, cellulase, GABA, c-di-GMP

## Abstract

Biofilm dispersal contributes to bacterial spread and disease transmission. However, its exact mechanism, especially that in the pathogen *Mycobacterium tuberculosis*, is unclear. In this study, the cellulase activity of the *M. tuberculosis* Rv0062 protein was characterized, and its effect on mycobacterial biofilm dispersal was analyzed by observation of the structure and components of Rv0062-treated biofilm in vitro. Meanwhile, the metabolite factors that induced cellulase-related biofilm dispersal were also explored with metabolome analysis and further validations. The results showed that Rv0062 protein had a cellulase activity with a similar optimum pH (6.0) and lower optimum temperature (30 °C) compared to the cellulases from other bacteria. It promoted mycobacterial biofilm dispersal by hydrolyzing cellulose, the main component of extracellular polymeric substrates of mycobacterial biofilm. A metabolome analysis revealed that 107 metabolites were significantly altered at different stages of *M. smegmatis* biofilm development. Among them, a decrease in gamma-aminobutyric acid (GABA) promoted cellulase-related biofilm dispersal, and this effect was realized with the down-regulation of the bacterial signal molecule c-di-GMP. All these findings suggested that cellulase promotes mycobacterial biofilm dispersal and that this process is closely associated with biofilm metabolite alterations.

## 1. Introduction

Biofilm is the structured bacterial communities encased in self-secreted extracellular polymeric substrates (EPSs). Compared with free-living planktonic cells, the bacteria in biofilms have more chances of survival in the adverse conditions of nutrient deprivation, dehydration, pH changes, and antimicrobial agents [1]. The EPS contains polysaccharides, lipids, nucleic acids, proteins, and other extracellular matrix components, which act as a diffusion barrier that not only prevents the access of antimicrobial agents to biofilm cells but also binds to the antimicrobial agents directly [2]. Therefore, biofilm cells are commonly 10–1000 times more tolerant of antibiotics compared with their planktonic cells [3].

Tuberculosis caused by *Mycobacterium tuberculosis* remains a dangerous infectious disease for human beings. In 2021, 6.4 million people were diagnosed as tuberculosis (TB) patients, and 1.6 million died from TB [4]. In recent years, *M. tuberculosis* and other mycobacteria, such as *M. bovis* BCG, *M. abscessus,* and *M. smegmatis*, were confirmed to form biofilm both in vitro [5,6,7,8,9] and in vivo [10,11]. They have a strong propensity to form biofilm when cultured in tubes or on plates, which can tolerate more than 50 times the minimal inhibitory concentration of isoniazid and rifampicin [6,7,8]. In patients, it was found that extracellular *M. tuberculosis* in human TB lesions formed large microbial communities similar to biofilms [12,13,14]. More recently, it was reported that the formation of biofilm in the lungs contributes to the virulence and drug tolerance of *M. tuberculosis* [15]. However, little is known about mycobacterial biofilm compared with well-studied bacteria, such as *Escherichia coli* and *Pseudomonas aeruginosa*.

Generally, biofilm development is a cyclic process that contains four stages: adhesion of planktonic cells, microcolony formation, biofilm maturation, and biofilm dispersal [16]. Dispersal is the last stage of the biofilm lifestyle at which sessile cells detach from the biofilm and transit to the planktonic mode of growth. By dispersion from old biofilm and recolonization at new sites, biofilm development enters a new cycle, and bacteria obtain more nutrients and energy from the host [16,17]. Environmental clues, such as nutritional deficiency and oxygen tension, and bacterial secretory enzymes, such as glycosidases, proteases, and autoinducers, were reported to be associated with dispersal [18].

Cellulose was revealed to be the key component of *M. tuberculosis* biofilm EPS in recent years. Using congo red staining, calcofluor white staining, and cellulase treatment, Trivedi et al. found that cellulose forms the biofilm skeleton of *M. tuberculosis*, which plays an important role in attaching bacteria at the surface, connecting the microcolonies, and recruiting planktonic bacteria in the biofilm [19]. In the *M. tuberculosis* genome, there are three cellulose-targeting genes that encode cellulose hydrolase, including *Cel6* (*rv0062*), *Cel12* (*rv1090*), and *CBD2* (*rv1987*) [20]. Among them, Rv0062 exists in most mycobacterium species and has been identified as a functional cellulase. It can hydrolyze both barley β-glucan and acid-swollen cellulose to release different gluco-oligosaccharides [21]. MSMEG_6752 is the ortholog of Rv0062 in *M. smegmatis* with 67% identities and 88% similarities. One study on MSMEG_6752 revealed that MSMEG_6752 can hydrolyze pre-formed biofilm to release oligosaccharides [22]. The overexpression of Rv0062 or MSMEG_6752 in *M. tuberculosis* inhibited the biofilm formation in vitro [15,22]. Overexpressing Rv0062 in *M. tuberculosis* also led to a lower bacterial load and less tissue damage in the lungs of infected mice [15]. All these findings suggested that cellulases are involved in mycobacterial biofilm formation. However, the exact function of cellulase in biofilm development and its mechanisms are still unknown. Since biofilm formation is an advantageous strategy for bacteria to survive in the environment and host, and cellulases are highly conserved in mycobacterium species, especially in pathogenic species, we suspect that cellulase contributes to biofilm dispersal by disrupting the cellulose skeleton in the natural biofilm life cycle. To clarify this suspicion, the enzymatic properties of Rv0062 and the effect of Rv0062 on mycobacterial biofilm dispersal were determined in this study. The cellulase expression in the model strain *M. smegmatis* mc^2^155 at different stages of biofilm development and the metabolic and signal clues that cause these alterations were also analyzed.

## 2. Results

### 2.1. Overexpression, Purification, and Cellulase Properties of Rv0062 Protein

**Figure 1:**
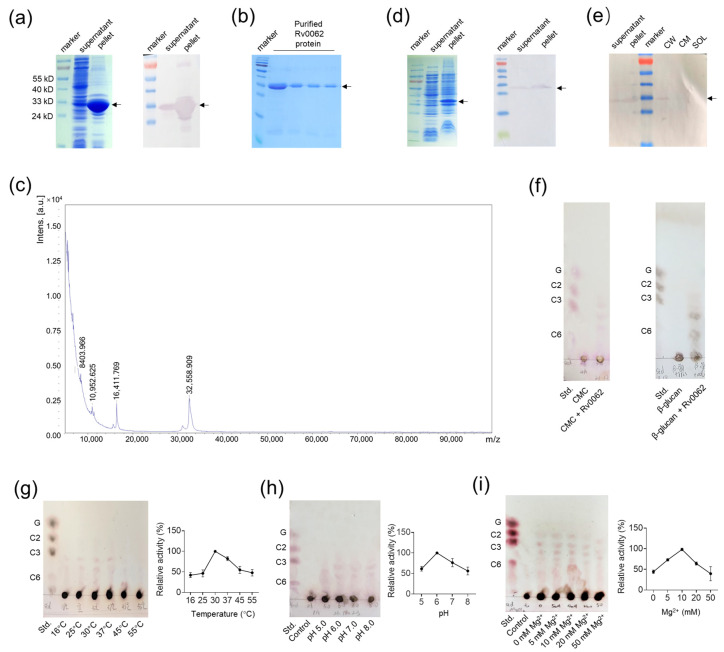
Overexpression, purification, and characterization of Rv0062 protein. (**a**) Detection of Rv0062 expression in the *E. coli* BL21(DE3) strain with SDS-PAGE and Western blot. (**b**) Detection of the purity of Rv0062 protein with SDS-PAGE. (**c**) Detection of the purity of Rv0062 protein with MALDI-TOF-MS analysis. (**d**) Detection of Rv0062 expression in the MS0062 strain with SDS-PAGE and Western blot. (**e**) Detection of the Rv0062 location in the MS0062 strain with Western blot. (**f**) TLC analysis of the products of Rv0062 catalytic reactions that were carried out withdifferent substrates. (**g**–**i**) TLC analysis of the products of Rv0062 catalytic reactions that were performed with CMC as substrate at different temperatures (**g**), pH values (**h**), and Mg^2+^ concentrations (**i**). The results were obtained from three dependent experiments (*n* = 3) and are shown as mean ± SEM. The arrows in (**a**,**b**,**d**,**e**) indicate the bands of Rv0062 protein. CW, cell wall; CM, cell membrane; SOL, soluble fractions; CMC, carboxymethyl cellulose; Std., standard; G, glucose; C2, cellobiose; C3, cellotriose; C6, cellohexose.

The coding sequence of the catalytic domain of the *M. tuberculosis* Rv0062 protein without the signal peptide was cloned into a pET16b vector and overexpressed in *E. coli* BL21(DE3) cells with an expected molecular weight of 32 kD (Figure 1a). Rv0062 was well purified with Ni^2+^ affinity chromatography, which was confirmed with SDS-PAGE (Figure 1b) and matrix-assisted laser desorption ionization time-of-flight mass spectrometry (MALDI-TOF-MS) analysis (Figure 1c). On the other hand, the coding sequence of the catalytic domain of Rv0062 was also cloned into a pVV2 plasmid and expressed in *M. smegmatis* mc^2^155 (Figure 1d), which was named strain MS0062. Subcellular localization of Rv0062 in MS0062 cells with differential centrifugation found that Rv0062 was mostly expressed in the cell wall rather than in the cell membrane and soluble fractions (Figure 1e). Meanwhile, MSVec, an *M. smegmatis* mc^2^155 strain harboring empty vector pVV2 [23], was used as a control strain in the following studies.

With the purified Rv0062 protein, the Rv0062 catalytic reactions were performed, and the products were analyzed using thin-layer chromatography (TLC). The results showed that Rv0062 had an endo-β-1,4-cellulase activity with a low specificity to substrates. It hydrolyzed both carboxymethyl cellulose (CMC) and β-glucan-releasing oligosaccharides (Figure 1f). It showed the highest enzyme activity toward CMC at 30 °C (Figure 1g) and pH 6.0 (Figure 1h). This optimum pH of Rv0062 was similar to that of the cellulases from other bacteria, but the optimum temperature of Rv0062 was relatively low [24,25]. In addition, Mg^2+^ contributes to the cellulase activity of Rv0062 with an optimum concentration of 10 mM (Figure 1i).

### 2.2. Cellulase Hydrolyzes the Cellulose in Mycobacterial Biofilm

**Figure 2:**
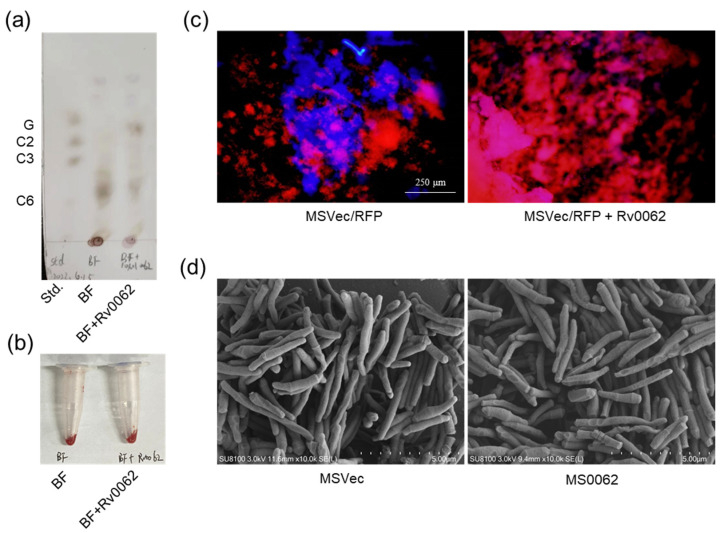
Rv0062 hydrolyzes the cellulose in mycobacterial biofilm. (**a**,**b**) Detection of the products of MSVec biofilms hydrolyzed by Rv0062 protein using TLC (**a**) and congo red staining (**b**). (**c**) The biofilm cellulose of MS0062/RFP bacteria after Rv0062 protein treatment was strained with calcofluor white and observed with a fluorescence microscope. The red color was from MS0062/RFP bacteria, and the blue color was from the calcofluor while-stained cellulose. (**d**) The morphology of MSVec and MS0062 cells was observed with a scanning electron microscope (HITACHI, Regulus8100, Tokyo, Japan). BF, biofilm; RFP, red fluoresce protein; MSVec, *M. smegmatis* harboring pVV2 empty vectors; MS0062, *M. smegmatis* overexpressing Rv0062 protein.

To clarify whether Rv0062 can hydrolyze the cellulose in mycobacterial biofilm, the pre-formed air–liquid layer biofilm of MSVec bacteria was hydrolyzed by Rv0062 protein, and the products were analyzed with TLC and congo red staining. The results showed that polysaccharides and oligosaccharides exist in mycobacterial biofilm. However, after treatment with Rv0062 protein, the polymerization degree of biofilm saccharides decreased, and some oligosaccharides were produced (Figure 2a). Congo red is a dye that can bind to cellulose and display dark red colors. Staining the biofilm with congo red showed that the dark red color became lighter in the Rv0062-treated biofilm compared with the biofilm without treatment (Figure 2b). To further confirm the disruptive effect of Rv0062 on biofilm cellulose, the plasmid pCG76-RFP, which expresses red fluorescence protein (RFP), was transformed into MSVec to construct the MSVec/RFP strain. The biofilms of MSVec/RFP formed on the coverslips were then treated with Rv0062 protein and stained with calcofluor white. Calcofluor white can recognize and bind to cellulose and chitin emitting blue fluorescence. The results showed that the cellulose skeleton that existed in the MSVec/RFP biofilm disappeared after Rv0062 protein treatment (Figure 2c). All these results indicated that Rv0062 protein can hydrolyze the cellulose in mycobacterial biofilm. Since Rv0062 has a low specificity on carbohydrate substrates, we suspect it may influence the integrity of bacterial cell walls. Therefore, the planktonic cells of the MS0062 and MSVec strains were collected and observed with a scanning electron microscope. However, no significant difference was observed on the bacterial surface between the two strains (Figure 2d), indicating that the cellulase activity of Rv0062 only affected the integrity of the biofilms but not the bacterial cell walls.

### 2.3. Cellulase Promotes the Dispersal of Mycobacterial Biofilm

**Figure 3:**
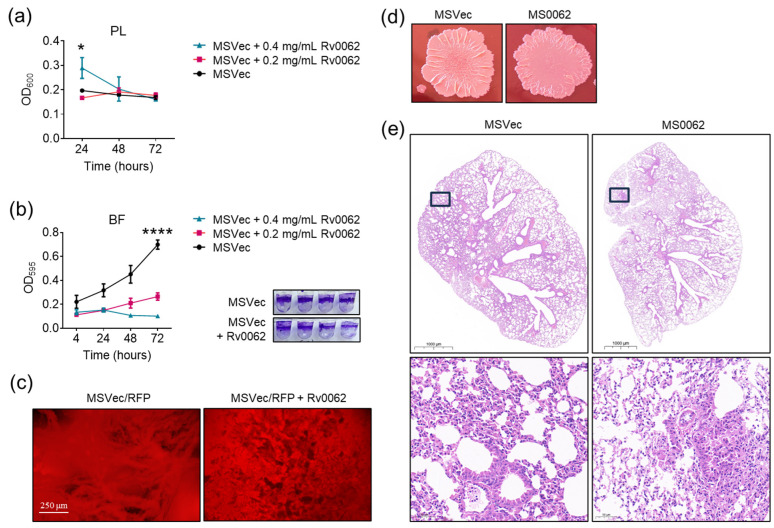
Rv0062 promotes biofilm dispersal by the hydrolyzation of cellulose. (**a**,**b**) The bacterial density of planktonic cells (**a**) and the remaining biofilm (**b**) of MSVec biofilm treated with Rv0062 protein in 96-well plates. The results were obtained from three dependent experiments (*n* = 3) and are shown as mean ± SEM. The difference between groups at a given time point was analyzed with a one-way ANOVA analysis with a post hoc Tukey’s multiple comparisons test (* *p* < 005; **** *p* < 0.0001). (**c**) The structure of the MSVec biofilm after Rv0062 treatment. (**d**) The colony morphology of MSVec and MS0062. (**e**) Lung histopathology of a mouse lung on day 7 post-infection with MSVec or MS0062. PL, planktonic; BF, biofilm; RFP, red fluoresce protein; MSVec, *M. smegmatis* harboring pVV2 empty vector; MS0062, *M. smegmatis* overexpressing Rv0062 protein.

To confirm whether Rv0062 can promote mycobacterial biofilm dispersal by its cellulase activity, Rv0062 protein was added to the pre-formed MSVec biofilm. The biofilm dispersal was analyzed by detecting the planktonic bacteria produced and the biofilm remaining in the wells. The results showed that there were significantly more planktonic bacteria in the wells to which 0.4 mg/mL Rv0062 was added than in the wells to which 0.2 mg/mL Rv0062 was added and in the wells without Rv0062 (Figure 3a). Correspondingly, the biofilm that remained in the wells was decreased after Rv0062 treatment (Figure 3b). To investigate the effect of Rv0062 on biofilm structure, the biofilm of MSVec/RFP grown at the bottom of well plates was treated with Rv0062 protein and observed after 24 h. It was found that Rv0062 treatment resulted in the formation of channels and holes in the mycobacterial biofilm (Figure 3c), which would facilitate bacteria to disperse from biofilms. In addition, the colony shape of MS0062 on agar plates was flatter than MSVec, with a trend of outward growth (Figure 3d). All these results indicated that Rv0062 stimulated the dispersal of mycobacterial biofilm in vitro. To clarify whether Rv0062-medicated biofilm dispersal leads to different results in host infection, the histopathology of lungs infected with MSVec and MS0062 was observed. The results showed that inflammation existed both in MSVec- and MS0062-infected mouse lungs; however, the inflammation was more serious and concentrated in MSVec-infected mice (Figure 3e). In MS0062-infected mice, the inflammation was relatively mild but distributed throughout the entire lung lobe. As Rv0062 has the ability to induce biofilm dispersal, we think the impaired inflammatory response was probably not due to the inhibitory effect of Rv0062 on biofilm formation but due to its promotional effect on bacterial spread in the whole lung. A more confident conclusion could be reached using large-scale animal experiments.

### 2.4. The Expression of Cellulase Is Upregulated during Mycobacterial Biofilm Dispersal

**Figure 4:**
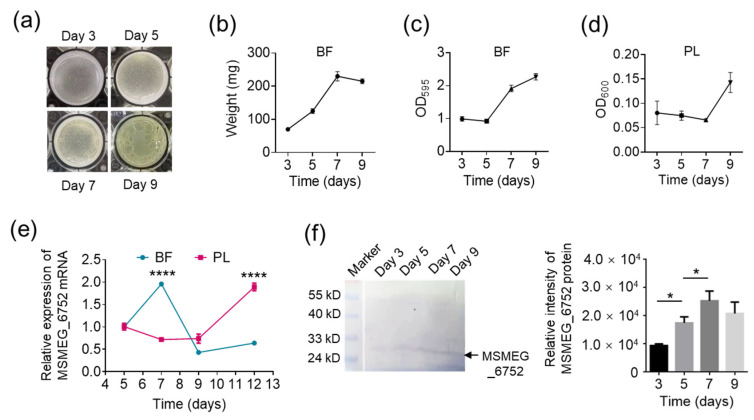
Cellulase is upregulated during the dispersal of mycobacterial biofilm. (**a**) The morphology of MSVec biofilm grown in 12-well plates at different time points. (**b**) The wet weight of MSVec biofilm formed in the air–liquid layer at different time points. (**c**) The crystal violet staining of MSVec biofilm formed on the sidewall of wells at different time points. (**d**) The bacterial density of planktonic cells at different time points. (**e**) The mRNA level of MSMEG_6752 in biofilm bacteria and planktonic cells at different stages of the biofilm life cycle. (**f**) The protein level of MSMEG_6752 in biofilm bacteria at different stages of biofilm development. The results in (**e**,**f**) were obtained from three dependent experiments (*n* = 3) and are shown as mean ± SEM. Statistical significance between different time points in biofilm or planktonic cells was determined using a one-way ANOVA analysis with a post hoc Tukey’s multiple comparisons test (* *p* < 0.05; **** *p* < 0.0001). PL, planktonic; BF, biofilm; MSVec, *M. smegmatis* harboring pVV2 empty vector.

Considering that biofilm dispersal is a proactive behavior of bacteria, we suspect that cellulase expression is well controlled by mycobacteria themselves at different stages of biofilm development. To clarify this suspicion, we analyzed the expression of cellulase MSMEG_6752 in the model strain *M. smegmatis* mc^2^155. The time points of the *M. smegmatis* biofilm life cycle in the 12-well plates were confirmed first. On days 3, 5, 7, and 9 after MSVec bacteria were inoculated in 12-well plates, the biofilms formed in the air–liquid layer (Figure 4a) were weighted, and the biofilms formed on the sidewall of the wells were stained with crystal violet. The results showed that the growth of both the air–liquid layer and sidewall biofilms increased rapidly from day 3 to day 7, but they all slowed down after day 7 (Figure 4b,c). Meanwhile, the number of planktonic cells increased from day 7 to day 9 (Figure 4d). These results indicated that the MSVec biofilm formed and matured during days 3 to 5 but began to disperse on day 7 in the 12-well plates. At these time points, we analyzed the expression of cellulase MSMEG_6752 in the MSVec biofilm. The results showed that the mRNA level of MSMEG_6752 in the biofilm bacteria was elevated from day 5 to day 7 when the biofilm was mature and prepared to disperse and dropped on day 9 when biofilm dispersion finished (Figure 4e). In contrast, the mRNA of MSMEG_6752 in planktonic cells maintained a low level in the progression of biofilm formation and maturation but reached a high level when biofilm dispersal was complete (Figure 4e). This high level of MSMEG_6752 expression in planktonic cells on day 9 may be from the biofilm-detached cells. To confirm these findings, we prepared Rv0062 polyclonal antibody by injecting C57BL/6 mice with purified Rv0062 protein. Western blot analysis revealed that the prepared antibody could recognize not only Rv0062 but also MSMEG_6752 (Supplementary Figure S1). Using this antibody, the expression of MSMEG_6752 in protein levels at different stages of biofilm development was analyzed. The results showed that MSMEG_6752 protein increased from day 3 to day 7 and remained at a relatively high level on day 9 (Figure 4f), which confirmed the upregulation of cellulase expression during biofilm dispersal at the protein level.

### 2.5. Metabolome of Mycobacterial Biofilm

**Figure 5:**
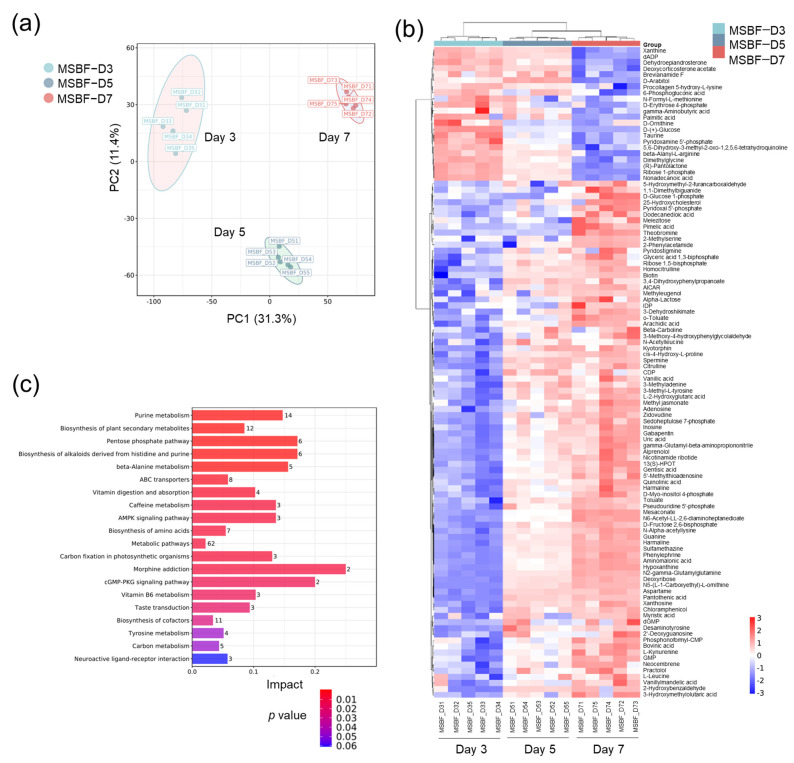
The metabolome of the mycobacterial biofilm. (**a**) PCA of the metabolites in *M. smegmatis* biofilm at different time points. (**b**) Significantly changed metabolites in *M. smegmatis* biofilm at different time points. (**c**) KEGG pathway analysis of significantly altered metabolites. The results are shown as mean ± SEM from 5 independent samples (*n* = 5) for each group. MSBF-D3, D5, and D7 represent *M. smegmatis* biofilm grown in 12-well plates for days 3, 5, and 7, respectively.

It was reported that nutrients and metabolites were the induction factors of biofilm dispersal [1,16]. Therefore, the metabolic profiles of the *M. smegmatis* mc^2^155 biofilm at different time points were analyzed with liquid chromatography (LC)–mass spectrometry (MS)/MS. A principal components analysis (PCA) of the identified metabolites showed that the metabolites from different groups were well separated (Figure 5a), indicating an obvious change in metabolites during biofilm development. From day 3 to day 7, the levels of 107 metabolites were significantly altered in the biofilm cells (Supplementary Table S1, Figure 5b). Some of them have been reported to be closely associated with biofilm development in bacteria, such as GABA [26,27], hypoxanthine [28], uric acid [29], and spermine [30]. The KEGG analysis of these metabolites found that they were rich in purine metabolites, the pentose phosphate pathway, beta-alanine metabolism, ABC transporters, etc. (Figure 5c).

### 2.6. A Decrease in GABA Induces the Cellulase Expression through the Down-Regulation of the c-di-GMP Level

**Figure 6:**
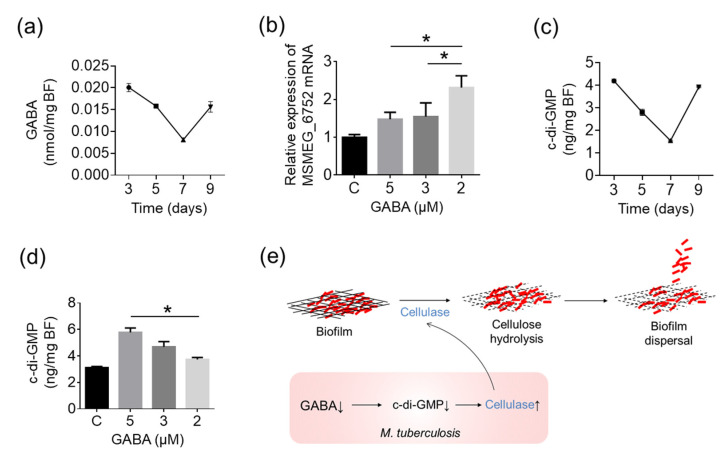
GABA decrease induces cellulase expression through the down-regulation of the c-di-GMP level. (**a**,**c**) The level of GABA and c-di-GMP in MSVec biofilm grown in 12-well plates at different time points. (**b**,**d**) MSMEG_6752 expression and c-di-GMP production in MSVec biofilm when the GABA concentration was dropped from 5 μM to 3 μM or 2 μM in the culture. The results in (**a**–**d**) were obtained from three dependent experiments (*n* = 3) and are shown as mean ± SEM. Statistical significance between groups in (**b**,**d**) was analyzed using a one-way ANOVA analysis with a post hoc Tukey’s multiple comparisons test (* *p* < 0.05). (**e**) The mechanism of mycobacterial biofilm dispersal induced by cellulase. MSVec, *M. smegmatis* harboring pVV2 empty vector.

Among the above significantly different metabolites, we focused on GABA, which has been reported to be involved in biofilm development. ELISA detection of the level of GABA in biofilm cells confirmed the results of the metabolome data. GABA declined with the biofilm maturation from day 3 to day 7 and reached the lowest level at day 7 when the biofilm began to disperse. On day 9, when dispersal was complete, the GABA level was recovered (Figure 6a). These results indicated that the decrease in GABA may have induced the dispersal of mycobacterial biofilm. To confirm this, an in vitro GABA decrease experiment was carried out to analyze the effect of GABA on cellulase expression. The results showed that the expression of MSMEG_6752 was significantly upregulated when the GABA level in the culture dropped from 5 μM to 2 μM (Figure 6b), suggesting that the decrease in GABA induced cellulase expression and consequently resulted in biofilm dispersal in mycobacteria.

c-di-GMP is an important intracellular signal involved in biofilm development in bacteria. Increased levels of c-di-GMP always lead to biofilm formation, whereas decreased levels of c-di-GMP generally induce biofilm dispersal, which has been observed in *E. coli*, *P. fluorescens*, *Salmonella enterica*, and other species [31,32,33,34]. Therefore, to clarify whether the cellulase upregulation and biofilm dispersal induced by GABA in mycobacteria was controlled by c-di-GMP, the c-di-GMP level in biofilm cells during biofilm development was detected. The results showed that the c-di-GMP level changed similarly with GABA at different stages of biofilm growth (Figure 6c). When exogenously added GABA was decreased in the culture, the c-di-GMP production in biofilm cells was reduced simultaneously (Figure 6d). These results indicated that the decrease in GABA led to the down-regulation of c-di-GMP, which consequently induced the secretion of cellulase and finally promoted mycobacterial biofilm dispersal (Figure 6e).

## 3. Discussion

As the key polysaccharide of the mycobacterial extracellular matrix, cellulose maintains the architecture of biofilm by forming fiber-like structures. In this study, we focused on the effect of secreted cellulase, a kind of glycosidase, on mycobacterial biofilm dispersal. Glycosidases, extracellular proteases, deoxyribonucleases, and chitinases are all reported to hydrolyze mature biofilm and lead to bacterial dismissions [16]. Among them, glycosidases are key enzymes because polysaccharides are the main component of biofilm EPSs in various bacterium species. For example, *P. aeruginosa* produces both alginate, a biofilm EPS polysaccharide, and alginate lyase, which can degrade alginate. Increased expression of the alginate lyase in *P. aeruginosa* led to alginate degradation and increased cell detachment [35]. Another well-studied biofilm dispersal-related glycosidase is dispersin B, which degrades poly-N-acetylglucosamine (PNAG), a biofilm matrix polysaccharide in *A. actinomycetemcomitans* [36]. The mutant of dispersin B fails to release cells from colonies into the medium, although its morphology looks similar to wild-type colonies [37]. In the present study, we confirmed that exogenously added *M. tuberculosis* cellulase Rv0062 resulted in the dispersal of pre-formed *M. smegmatis* biofilm. In addition, the expression of *M. smegmatis* cellulase MSMEG_6752 was increased when biofilm dispersal occurred. All these findings suggested that cellulase played a role in maintaining the life cycle of mycobacterial biofilm and contributed to bacterial spread during infections.

To clarify the factors that induce the upregulation of cellulase expression in biofilm dispersal, we studied the metabolites in biofilm cells. Nutrients and metabolites are extrinsic factors that promote biofilm dispersal. Unfavorable or favorable environmental conditions, such as a sudden increase or decrease in carbon and nitrogen sources or limited oxygen, may stimulate bacteria to disperse from biofilm [38,39,40]. Meanwhile, the metabolites from host cells, such as taurocholic acid and NO [41,42], and the metabolites from bacteria themselves, such as unsaturated fatty acids, phenazine, and glycopeptidolipid [43,44,45], are also reported to promote bacterial detachment from biofilm. Here, to discover the metabolites related to mycobacterial biofilm dispersal, the metabolome of biofilm cells was analyzed with LC-MS/MS. It was found that 107 metabolites were significantly different at various stages of the biofilm life cycle. We focused on GABA, a metabolite that was significantly decreased during mycobacterial biofilm dispersal. GABA has been reported to be produced by many bacteria, including *P. aeruginosa*, *P. fluorescens*, *E. coli*, etc. [46,47,48]. It acts as a communication molecule between bacteria or between bacteria and their host. Studies on *P. aeruginosa* and *P. fluorescens* revealed that pre-treated bacteria with exogenously added GABA in their growth phase reduced biofilm formation in microwells [46,47]. However, the effect of GABA on biofilm dispersal is still unknown. Our results in this study first confirmed the existence of GABA in mycobacterium species and its function in mycobacterial biofilm development. As a metabolic signal, the GABA level decreased with the maturation of the biofilm, which promoted cellulase expression and consequently led to biofilm dispersal. More studies are needed to understand the production pathway of mycobacterial GABA and its regulations in the future.

Moreover, to discover the mechanism underlying GABA-induced cellulose upregulation, the reported regulatory systems of bacterial biofilm dispersal must be studied. Quorum-sensing (QS) is the most well-studied regulatory system, especially in Gram-negative species. A wide range of chemical classes, such as autoinducing peptides, acyl-homoserine lactones, non-species-specific autoinducer 2, and cis-unsaturated fatty acids, are all QS signaling molecules [1]. Another system is the nucleotide-based second messenger signaling pathway, in which c-di-GMP is the core signal molecule. c-di-GMP was first discovered as an activator of cellulose synthesis in *Acetobacter xylinus* by Benziman and coworkers in 1987 [49], indicating its close association with cellulose metabolism in bacteria. In general, a high c-di-GMP level promotes biofilm formation by enhancing the biosynthesis of adhesins and matrix polysaccharides, whereas a low c-di-GMP level leads to biofilm dispersal [50,51]. However, the relationship between c-di-GMP and cellulose metabolism has not been reported so far. In this study, c-di-GMP was found to be decreased during mycobacterial biofilm dispersal, and this decrease was in response to GABA alterations. As the GABA decrease was the metabolic factor that induced cellulase expression, c-di-GMP would act as a message molecule during this GABA-induced biofilm dispersal. However, identifying how c-di-GMP responds to GABA needs to be studied further. In *M. smegmatis*, a dual-active enzyme, DcpA, was found to be involved in c-di-GMP biosynthesis. Alteration in the DcpA level affected the cell length and colony morphology of *M. smegmatis* [52]. Orthologs of DcpA are also present in *M. tuberculosis* and other mycobacterium species with a high degree of sequence similarity and domain architecture [53]. Studies on these c-di-GMP synthesis-related enzymes will provide new clues for the clarification of signal pathways that are involved in mycobacterial biofilm dispersal.

Noticeably, the fast-growing and non-pathogenic *M. smegmatis* cannot represent various mycobacterium species in biofilm-forming abilities. Including other mycobacteria, such as *M. tuberculosis*, would strengthen the generalizability of these findings on mycobacterial biofilm dispersal. Also, it would be better if the effect of cellulase on biofilm dispersal could be observed directly in vivo. In addition, the mechanisms of GABA regulating biofilm dispersal, including the reason for the GABA decrease and the pathway in which GABA mediates c-di-GMP levels, are not clarified and are worthy of study in the future.

## 4. Materials and Methods

### 4.1. Overexpression of Rv0062 in E. coli and M. smegmatis Strains

The coding sequence of Rv0062 protein (89-380 amino acids) was amplified using PCR amplification with the primers *rv0062*-F: 5′ TTCATATGAACCCGCTGGCC GGAAAG 3′ and *rv0062*-R: 5′ TTGGATCCCTACTGGCCGGCGTTGTG 3′. The PCR product was cloned into a pJET1.2 vector for sequencing and then subcloned into the BamHI and NdeI sites of pET16b and pVV2 vectors. The pET16b-*rv0062* plasmid was transformed into *E. coli* BL21(DE3) cells (Merck, Catalog No. 69450-M) to generate strain EC0062, which was grown in LB broth containing 100 μg/mL ampicillin. The expression of Rv0062 protein was induced by adding 1 mM IPTG in the culture and growing the bacteria at 37 °C for another 3.5 h, which was finally detected with Western blot using an anti-His-tag antibody.

The pVV2-*rv0062* plasmid was transformed into *M. smegmatis* mc^2^155 cells (ATCC, Catalog No.700084) to generate the recombinant strain MS0062. MSVec, the *M. smegmatis* mc^2^155 strain carrying empty vector pVV2, was constructed in our previous study [31]. MS0062 and MSVec were cultured in LBT (LB broth with 0.05% Tween 80) containing 25 μg/mL kanamycin. To express the Rv0062 protein, MS0062 cells were cultured at 37 °C for 24 h. The cells were collected, and different cellular fractions, including the cell wall (CW), cell membrane (CM), and soluble fraction of cytoplasm (SOL), were separated with differential centrifugation as described previously [54]. The expression of Rv0062 in these fractions was analyzed with Western blot using anti-His antibody. In addition, pCG76-RFP, a plasmid expressing red fluorescence protein, was transformed into MS0062 and MSVec to construct MS0062/RFP and MSVec/RFP, respectively.

### 4.2. Purification of Rv0062 Protein

The EC0062 cells were collected with centrifugation and broken with sonification in a lysis buffer (20 mM Tris-HCl, pH 8.0, 500 mM NaCl, 20% glyceride, 1 mM PMSF). The lysate was then centrifuged at 12,000× *g* for 20 min, and the supernatant was applied to a 1.0 mL column volume of Ni-NTA Superflow (Qiagen, Venlo, The Netherlands) to purify the Rv0062 protein with affinity chromatography. The column was washed with 20 mL washing buffer (lysis buffer with 40 mM imidazole), and then the Rv0062 protein was eluted with 10 mL elution buffer (lysis buffer with 200 mM imidazole). The purity of the Rv0062 protein in the solution was confirmed with SDS-PAGE and MALDI-TOF-MS analysis. For the following enzymatic reactions and function studies, the purified Rv0062 protein was concentrated using ultrafiltration with a 10 kDa cutoff filter (Millipore, Burlington, MA, USA) and finally resolved in 20 mM Tris-HCl (pH 8.0).

For the MALDI-TOF-MS analysis, the purified Rv0062 protein was desalted using ultrafiltration with a 10 kDa cutoff filter and then resolved in 0.1% trifluoroacetic acid solution. An aliquot (1.5 μL) of this protein solution (0.5 mg/mL) was spotted on the target plate that had been covered using 0.5 μL acetonitrile: 0.1% trifluoroacetic acid (3:7, *v*/*v*) solution saturated sinapinic acid. Spectra were acquired with a MALDI-TOF mass spectrometer (New ultrafleXtreme MALDI-TOF/TOF; Bruker Daltonik, Bremen, Germany) from Na+ adduct ions using the linear mode. The spectrum range was set from *m*/*z* 5000 to 100,000.

### 4.3. Cellulase Activity

The cellulase activity of the Rv0062 protein was analyzed by performing enzymatic reactions with substrate CMC (Aladdin, Shanghai, China) or β-glucan (Sigma, St. Louis, MO, USA). The rection contains 60 μL 1% CMC or 4% glucan (dissolved in 20 mM sodium phosphate buffer, pH 6.0) and 20 μL 5 mg/mL purified Rv0062 protein. The control reaction was performed with substrates and 20 uL of 20 mM Tris-HCl (pH 8.0). The mixture was incubated at 37 °C for 1 h. For an optimum pH analysis, CMC was dissolved in 20 mM sodium phosphate buffer at different pH values (5.0, 6.0, 7.0, and 8.0). For an optimum temperature analysis, the reactions were carried out at different temperatures (16, 25, 30, 37, 45, and 55 °C). To confirm the effect of ions on the cellulase activity of Rv0062, the reactions were performed in the presence of different concentrations of Mg^2+^ (5, 10, 20, and 50 mM).

### 4.4. TLC Analysis

The products of enzymatic reactions were centrifuged at 2000× *g* for 15 min, and 4 μL supernatant was spotted on TLC Silica Gel 60 F254 plates (Merk, Darmstadt, Germany). The oligosaccharide mixture was prepared by mixing equal volumes of 10 mg/mL glucose (G), cellobiose (C2), cellotriose (C3), and cellohexose (C6), which was spotted on the plates as a standard. TLC analysis was performed in a solvent system of butanol–acetic acid–distilled water (2:1:1, *v*/*v*/*v*). The products were finally visualized by spraying 10% sulfuric acid in ethanol on the plates and heating the plates at 120 °C for 3–5 min.

### 4.5. Biofilm Growth

To grow the biofilm in well plates, the wild-type *M. smegmatis* mc^2^155, MSVec, MS0062, MSVec/RFP, or MS0062/RFP was cultured in LBT containing related antibiotics for 48 h. The cells were then diluted in M63 culture (76 mM (NH_4_)_2_SO_4_, 500 mM KH_2_PO_4_, 5.8 μM FeSO_4_, 0.05% Tween 80, 2% glucose, pH 7.0) containing related antibiotics. The optical density at 600 nm (OD_600_) of the diluted bacteria was about 0.1–0.15. The diluted bacteria were incubated in 96- or 12-well plates and grown at 37 °C for 3, 5, 7, 9, or 12 days in a static state to allow for biofilm formation and dispersal. If necessary, the glass coverslips were placed in 12-well plates. The biofilm in the air–liquid layer was collected at different time points for weighting, congo red staining, RNA isolation, bacterial protein preparation, GABA detection, and performing enzymatic reactions. The biofilm formed on the sidewall of the wells was stained with crystal violet. The biofilm formed at the bottom of the well or coverslip was observed with a fluorescence microscope or stained with calcofluor white.

### 4.6. Hydrolysis of Biofilm by Rv0062

The air–liquid layer biofilm of MSVec grown in 12-well plates for 5 days was collected. The in vitro Rv0062-catalyzed hydrolysis reaction contained 100 mg biofilm (wet weight), 40 μL 20 mM sodium phosphate buffer (pH 6.0), and 20 μL 5 mg/mL purified Rv0062 protein. The reaction without adding Rv0062 protein was set as a control. The cellulose remaining in the biofilm was measured with congo red staining, and the released oligosaccharides from the biofilm were analyzed with TLC.

For the in situ hydrolysis of biofilm cellulose with Rv0062, the biofilms of MSVec and MSVec/RFP were grown in 96-well plates for 5 days, respectively. The culture in the well was changed with fresh M63 containing 200 μg/mL or 400 μg/mL purified Rv0062 protein. After incubation at 37 °C for 4, 24, 48, and 72 h, the OD_600_ of the planktonic bacteria of MSVec in the 96-well plates were monitored with a microplate reader (Thermo Scientific Multiskan Ascent, Waltham, MA, USA), and the biofilm formed on the sidewall of the wells was measured with crystal violet staining. To clarify the effect of Rv0062 hydrolyzation on the biofilm structure, the biofilm of MSVec/RFP was grown in 12-well plates, treated with Rv0062, and observed with a fluorescence microscope after 24 h of treatment. To observe the cellulose remaining in the biofilm structure, the coverslips were placed in the wells during the above processes and stained with calcofluor white in situ.

### 4.7. Congo Red Staining

The air–liquid layer biofilm was added to 200 μL 20% congo red and incubated at 37 °C for 2 h. The stained biofilm was collected with centrifugation and washed with phosphate-buffered saline (PBS, obtained from Solarbio, Beijing, China) three times. The colors of the Rv0062-treated and untreated biofilms were compared.

### 4.8. Crystal Violet Staining

The biofilm formed on the side wall of the 96-well plates was washed with PBS three times and then stained with 200 μL 0.1% crystal violet reagent (Solarbio, Beijing, China) for 15 min at room temperature. After washing with PBS, the crystal violet binding on the biofilm was resolved in 200 μL 95% ethanol for 2 min. The solution was centrifuged at 6000× *g* for 2 min, and the OD_595_ of the supernatant was read with a microplate reader.

### 4.9. Isolation of Bacterial RNA and RT-qPCR

The air–liquid layer biofilm grown in the 12-well plates was collected, washed with PBS, and broken with an automatic homogenizer with 0.1 mm beads. The bacterial total RNA was isolated with the RNAiso plus reagent (TaKaRa, Otsu, Japan) from the lysate according to the manufacturer’s instructions. After reading the absorbance at 260 nm, 1 µg of isolated RNA was immediately reverse-transcribed to cDNA using the PrimeScriptTM RT reagent kit with gDNA Eraser (TaKaRa). The expression of MSMEG_6752 was analyzed using qPCR with the TB Green^®^ Premix Ex Taq TM II kit (TaKaRa) and the primers MSMEG_6752-F: GACGTGTCGA TGACGTAGTGC and MSMEG_6752-R: TGGGTCAACGCCGAGGACATG. For the control, 16S rRNA was selected, which was amplified with primers 16S rRNA-F: GAAGCGCAAGTGACGGTATGTG and 16S rRNA-R: ACGCCCACAGTTAAGCTGTGAG.

### 4.10. Western Blot

The protein samples were firstly separated with SDS-PAGE, transferred to a nitrocellulose membrane, and then incubated with the corresponding antibodies. For the detection of the expression of Rv0062 protein fused with a His-tag at the N-terminal, the protein bands were incubated with anti-His antibody (anti)-polyhistidine clone HIS-1 antibody (Sigma, St. Louis, MO) and finally visualized in BCIP/NBT solution. To detect the expression of MSMEG_6752 in MSVec at different stages of biofilm development, the same amount of total protein (40 μg) was applied on SDS-PAGE, and the protein bands in the membrane were detected with anti-Rv0062 polyclone antibody prepared by ourselves, which were finally visualized with ECL reagents (Advansta, San Jose, CA, USA). The protein bands on the Western blot were quantified into relative expressions using Image lab 4.0.1 software.

### 4.11. Infection of Mice

C57BL/6 mice were infected with MS0062 and MSVec using an aerosol generator (Kangjie Instrument, Liaoyang, China) at 5 × 10^9^ CFU/day/mouse for 4 days as described in our previous study [23]. On day 7 post the first day of infection, the mice were sacrificed, and the lung tissues were collected sterilely for lung histopathology.

### 4.12. Lung Histopathology

The lung tissues obtained from the infected mice were fixed with 10% formalin in PBS. Pathological slides were then prepared and stained using H&E as described in the report [55] The slides were finally observed with CaseViewer 2.4 software (3DHISTECH Ltd. Budapest, Hungary).

### 4.13. Calcofluor White Staining

The cellulose of the biofilm on the coverslips was washed with PBS three times to remove the planktonic bacteria. The coverslips were then placed at 60 °C for 1 h to fix the biofilm and then stained with 0.1 mg/mL calcofluor white for 20 min in a dark room. After being washed with PBS three times, the biofilm was observed with a fluorescence microscope (SUNNY ICX41, Yuyao, China).

### 4.14. Analysis of Bacterial Metabolites in Biofilm LC/MS-MS

One hundred micrograms of the air–liquid layer biofilms of wild-type *M. smegmatis* grown in 12-well plates for 3, 5, and 7 days were collected and quick-frozen in liquid nitrogen. To isolate metabolites, the biofilm was thawed and added to 100 mg glass bead and 1 mL 50% cold methanol–water and vortexed for 30 s. This process was repeated two times, and then the samples were centrifuged for 10 min at 12,000× *g* at 4 °C. The supernatant was transferred to a new tube and concentrated and dried. The metabolites were finally resolved in a 2-amino-3-(2-chloro-phenyl)-propionic acid (4 ppm) solution prepared with 50% methanol–water for LC-MS detection.

The LC analysis was performed on a Vanquish UHPLC System (Thermo Fisher Scientific, Waltham, MA, USA). Chromatography was carried out with an ACQUITY UPLC ^®^ HSS T3 (150 × 2.1 mm, 1.8 µm) (Waters, Milford, MA, USA). The column was maintained at 40 °C. The flow rate and injection volume were set at 0.25 mL/min and 2 μL, respectively. For LC-ESI (+)-MS analysis, the mobile phases consisted of 0.1% formic acid in acetonitrile (*v*/*v*) (C) and 0.1% formic acid in water (*v*/*v*) (D). Separation was conducted under the following gradient: 0~1 min, 2% C; 1~9 min, 2~50% C; 9~12 min, 50~98% C; 12~13.5 min, 98% C; 13.5~14 min, 98~2% C; and 14~20 min, 2% C. For LC-ESI (-)-MS analysis, the analytes were carried out with acetonitrile (A) and 5 mM ammonium formate (B). Separation was conducted under the following gradient: 0~1 min, 2%A; 1~9 min, 2~50%A; 9~12 min, 50~98%A; 12~13.5 min, 98%A; 13.5~14 min, 98~2%A; and 14~17 min, 2%A. Mass spectrometric detection of metabolites was performed on Orbitrap Exploris 120 (Thermo Fisher Scientific, USA) with an ESI ion source. Simultaneous MS1 and MS/MS (Full MS-ddMS2 mode, data-dependent MS/MS) acquisition was used. The parameters were as follows: sheath gas pressure, 30 arb; aux gas flow, 10 arb; spray voltage, 3.50 kV and −2.50 kV for ESI (+) and ESI (–), respectively; capillary temperature, 325 °C; MS1 range, *m*/*z* 100–1000; MS1 resolving power, 60,000 FWHM; number of data-dependent scans per cycle, 4; MS/MS resolving power, 15,000 FWHM; normalized collision energy, 30%; and dynamic exclusion time, automatic.

### 4.15. GABA Detection

The air–liquid layer biofilms of MSVec grown in 12-well plates for 3, 5, 7, and 9 days were harvested. The level of GABA in biofilm cells was detected with a GABA ELISA kit (Jiangsu Meimian Industrial Co., Ltd., Nanjing, China).

### 4.16. Growth of Biofilm In Vitro with a Decrease in the GABA Level

To observe the effect of a decrease in GABA on biofilm dispersal, MSVec were cultured in M63 broth with 5 μM GABA for 3 days in 12-well plates, which was then changed with fresh M63 broth containing 2, 3, or 5 μM GABA. The bacteria grown without GABA was set as a control. After 24 h, the air–liquid layer biofilm was weighted and collected for the detection of MSMEG_6752 mRNA and c-di-GMP levels.

### 4.17. c-di-GMP Detection

The air–liquid layer biofilm formed in 12-well plates on days 3, 5, 7, and 9 and the air–liquid layer biofilm exposed to a decrease in GABA levels were collected. The c-di-GMP level in the biofilm cells was then determined with a c-di-GMP ELISA kit (Jiangsu Meimian Industrial Co., Ltd.).

### 4.18. Statistical Analysis

Statistical analyses were performed using GraphPad Prism 6.01 software. A one-way ANOVA analysis with a post hoc Tukey’s multiple comparisons test was used to determine the difference between groups.

## 5. Conclusions

In this study, the enzyme-promoted biofilm dispersal and its molecular mechanism in mycobacteria were studied. As a secretory hydrolase, cellulase promoted mycobacterial biofilm dispersal by hydrolyzing the main biofilm matrix component, cellulose. In this process, mycobacteria sensed the decrease in the bacterial metabolite GABA and upregulated the cellulase expression through the second-message molecule c-di-GMP.

## Data Availability

All data generated or analyzed during this study are included in this published article and its supplementary information files.

## Supplementary Materials

The following supporting information can be downloaded at: https://www.mdpi.com/article/10.3390/ijms25021051/s1.
