# Supplementary figures and images for: Cellulase Promotes Mycobacterial Biofilm Dispersal in Response to a Decrease in the Bacterial Metabolite Gamma-Aminobutyric Acid
Source: Int J Mol Sci. 2024 Jan 15;25(2):1051. doi: 10.3390/ijms25021051 (PMC10816823; doi:10.3390/ijms25021051)

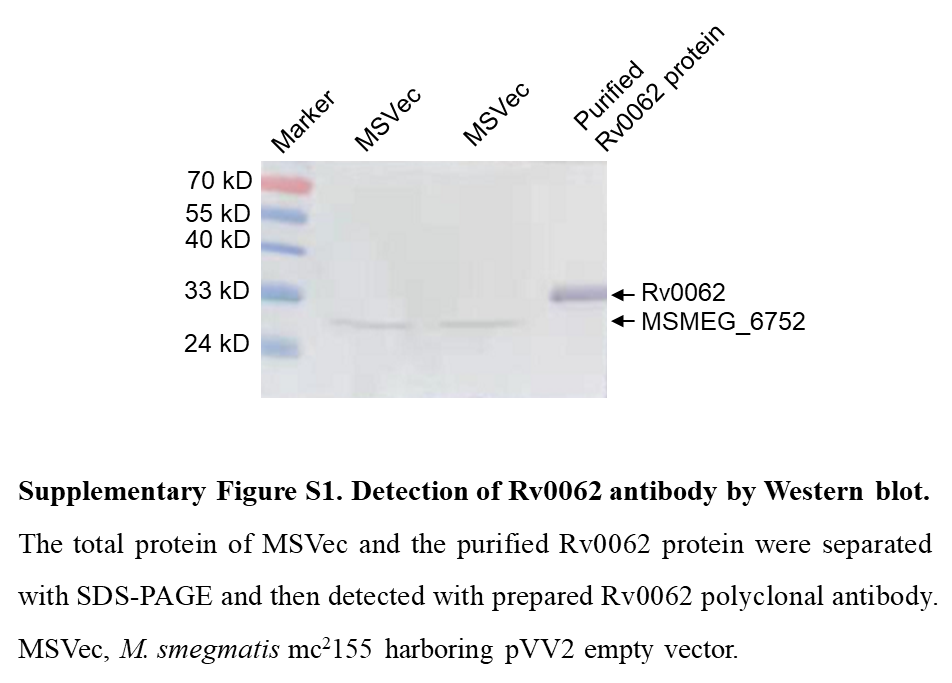

Supplement: Supplementary file 1 [file ijms-25-01051-s001.zip › Supplementary Figure S1.tif]
